# SARS-CoV-2 remodels the landscape of small non-coding RNAs with infection time and symptom severity

**DOI:** 10.1038/s41540-024-00367-z

**Published:** 2024-04-17

**Authors:** Julia Corell-Sierra, Joan Marquez-Molins, María-Carmen Marqués, Andrea Gabriela Hernandez-Azurdia, Roser Montagud-Martínez, María Cebriá-Mendoza, José M. Cuevas, Eliseo Albert, David Navarro, Guillermo Rodrigo, Gustavo Gómez

**Affiliations:** 1 Institute for Integrative Systems Biology (I2SysBio), CSIC – University of Valencia, 46980 Paterna, Spain; 2 Microbiology Service, Clinic University Hospital, INCLIVA Biomedical Research Institute, 46010 Valencia, Spain; 3 Department of Microbiology, School of Medicine, University of Valencia, 46010 Valencia, Spain; 4 Present Address: Department of Plant Biology, Uppsala BioCenter, Swedish University of Agricultural Sciences and Linnean Center for Plant Biology, Uppsala, Sweden

**Keywords:** Molecular biology, Computational biology and bioinformatics, SARS-CoV-2

## Abstract

The COVID-19 pandemic caused by the coronavirus SARS-CoV-2 has significantly impacted global health, stressing the necessity of basic understanding of the host response to this viral infection. In this study, we investigated how SARS-CoV-2 remodels the landscape of small non-coding RNAs (sncRNA) from a large collection of nasopharyngeal swab samples taken at various time points from patients with distinct symptom severity. High-throughput RNA sequencing analysis revealed a global alteration of the sncRNA landscape, with abundance peaks related to species of 21-23 and 32-33 nucleotides. Host-derived sncRNAs, including microRNAs (miRNAs), transfer RNA-derived small RNAs (tsRNAs), and small nucleolar RNA-derived small RNAs (sdRNAs) exhibited significant differential expression in infected patients compared to controls. Importantly, miRNA expression was predominantly down-regulated in response to SARS-CoV-2 infection, especially in patients with severe symptoms. Furthermore, we identified specific tsRNAs derived from Glu- and Gly-tRNAs as major altered elements upon infection, with 5’ tRNA halves being the most abundant species and suggesting their potential as biomarkers for viral presence and disease severity prediction. Additionally, down-regulation of C/D-box sdRNAs and altered expression of tinyRNAs (tyRNAs) were observed in infected patients. These findings provide valuable insights into the host sncRNA response to SARS-CoV-2 infection and may contribute to the development of further diagnostic and therapeutic strategies in the clinic.

## Introduction

The outbreak of pneumonia that began at the end of 2019 in Wuhan (China) was later found to be associated with the emergence of a novel coronavirus. This novel virus has been formally named as severe acute respiratory syndrome coronavirus 2 (SARS-CoV-2)^1^. This is a positive-sense single-stranded RNA virus with a genome length of about 30 kb, and it is the causative agent of the coronavirus disease 2019 (COVID-19) that has provoked a global public health crisis. Such a disease is characterized by respiratory distress, fever, cough, fatigue, pneumonia, and muscle pain^2–4^. Although diverse studies have focused on elucidating the pathogenesis and pathophysiology of COVID-19^5^, our knowledge about the transcriptomic remodeling produced upon infection remains yet in a conundrum, especially if we focus on the changes occurred in the non-coding layer^6^. Hence, the identification of cellular factors involved in this RNA-based network is essential to better understand the mode of action of the virus and consequently to develop further antiviral strategies^7^.

Increasing evidence suggests the existence of a close relationship between the SARS-CoV-2 infection and the altered accumulation of endogenous small non-coding RNAs (sncRNAs, or simply sRNAs)^6,8–11^. Of note, sncRNAs are pervasive in all kingdoms of life (from prokaryotes to eukaryotes) and they are involved in the regulation of gene expression^12^. Typically, sncRNAs comprise RNA molecules of length smaller than 50 nt. These include microRNAs (miRNAs), PIWI-interacting RNAs (piRNAs)^13,14^, and emerging sncRNAs such as those derived from transfer RNA (tsRNAs)^15,16^, small nuclear RNAs (snsRNAs) and small nucleolar RNAs (sdRNAs)^17^ and trimmed forms of miRNAs dubbed as tiny RNAs (tyRNAs)^18^. Although the non-canonical sncRNAs were initially considered as degradation products of the RNA metabolism, their functional roles are recently being unveiled, thereby their study is gaining importance^12^.

Regarding their biogenesis, canonical miRNAs (in vertebrates) are generated from structured (hairpin) RNA precursors by ribonuclease (RNase) III enzymes (Drosha and Dicer)^19^. In short, the pri-miRNA transcript is “*cropped*” by the nuclear Microprocessor complex (Drosha/DGCR8), releasing a precursor miRNA (pre-miRNA) that is exported to the cytoplasm and cleaved by Dicer^19^. In contrast, piRNAs arise from linear RNA precursors independently of Dicer and Drosha activity^20^. Considering tsRNAs, there are two main classes: tRNA-derived fragments (termed tRFs), which are theoretically processed by the RNA interference machinery in the D- or T-loops, and tRNA halves, which are 30–35 nt fragments generated by cleaving the anticodon loop by diverse RNases (e.g., Angiogenin in mammals)^15,16^. The generation of mature sdRNAs is predominantly based on the classic miRNA biogenesis pathway, involving Drosha and Dicer. The sdRNA length slightly varies depending on whether the parental snoRNA belongs to the C/D box family ( ~ 27 nt) or to the H/ACA-box family (17-19 nt)^17^. Moreover, tyRNAs are derived from the activity of several 3’-to-5’ exonucleases capable of trimming Argonaute (AGO)-associated full-length miRNAs into 14-nt or shorter RNA molecules^18^. In humans, trimming occurs in a manganese-dependent manner, but independently of the guide sequence and the AGO complex^18^.

The function of miRNAs and piRNAs is based on the complementarity with their RNA and DNA targets, leading to RNA silencing, translation repression, or transcriptional repression via the AGO/PIWI family proteins^21^. The accumulation of tsRNAs has been noticed in stress conditions, such as starvation, cancer, and virus infection^16^. Early studies showed the association of diverse tsRNAs with AGO and PIWI proteins, indicating that they can enter the RNA interference pathway, in both animal^22,23^ and plant^15^ cells. In addition, a close interaction between tsRNAs and diverse RNA-binding proteins and ribosomes has been described in mammals^16^. sdRNAs function as a molecular guide of mainly AGO2. Perfect complementarity by sdRNAs leads to target RNA cleavage, while an imperfect binding results in translation repression^17^. Regarding the function of tyRNAs, recent studies suggest that they may participate in an alternative RNA cleavage route by increasing the slicing activity of human AGO3^24^.

In this work, we performed an integrative study of the sncRNA landscape by analyzing multiple clinical samples collected from patients with severe and moderate symptomatology at two different stages of infection. Our study goes beyond previous work that described an association between SARS-CoV-2 infection and an imbalance in host sncRNAs^25^ with more statistical power and clinical implication. Our results provide evidence that SARS-CoV-2 infection induces, in addition to the previously described alterations in the accumulation of host miRNAs and tsRNAs, a significant imbalance of certain sdRNAs and tyRNAs, thereby providing novel insight about the mode of action of SARS-CoV-2 and allowing to recognize the involvement of this regulatory RNA layer in the infection process.

## Results

### sncRNA quantification and classification

**Fig. 1:**
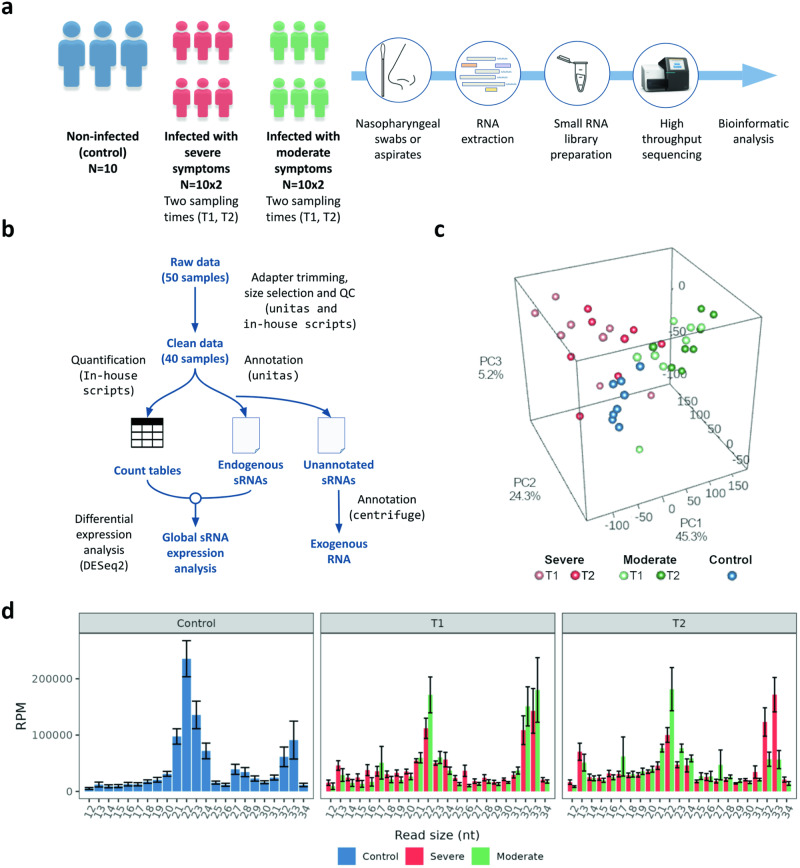
Experimental design and bioinformatic analysis. **a** Overview of the experimental design, in which sample acquisition is followed by extraction, preparation, and sequencing of sncRNAs. **b** Bioinformatics pipeline for analysis of sncRNA sequencing data. **c** Principal component analysis based on sRNA accumulation. **d** Histogram showing the relative accumulation (and distribution) of the total clean reads of sRNAs ranging from 12 to 34 nt from the libraries analyzed. Controls and different patient groups are represented with colors. Error bars correspond to standard errors. RPM reads per million. Error bars represent mean ± standard error of the mean.

Clinical samples from nasopharyngeal (swabs or aspirates) samples were collected from 20 SARS-CoV-2 infected patients at two different time points (T1 and T2). The infection was determined by reverse transcription quantitative polymerase chain reaction (RT-qPCR) in the hospital. T1 corresponds to an initial time at which the first symptoms appear (about 5-7 days post-infection), while T2 corresponds to a late time (about 19–21 days post-infection, resulting in a difference of 14 ± 4 days). In addition, nasopharyngeal swabs from 10 patients that resulted negative in SARS-CoV-2 infection were also collected as a control group. COVID-19 patients were clinically classified (according to symptom intensity) as moderate and severe (Fig. 1a and Supplementary Table 1). To obtain a global expression profile of the human sncRNAs, RNA was extracted from samples (enriched for small RNAs) and was subject to Illumina high-throughput sequencing. Raw data were processed as depicted in Fig. 1b. Low-quality libraries (two for each group of study) were discarded, which resulted in 40 libraries for further analysis. A total of 396,323,879 high-quality reads were obtained (Supplementary Table 2). Associations between sncRNA profiles (considering all types of samples and their biological replicates) were assessed by principal component analysis (PCA). The percentage of the total variance explained by the first three PCs was 74.8% (Fig. 1c). The different groups of study regarding outcome and time of infection were predominantly separated according to disease status (severe and moderate) (Supplementary Figure 1). We did not observe in our study associations between sRNA expression and sex or sampling intervals (Supplementary Fig. 1). In contrast, as it is well established^2,26^, severe cases were clearly related to elderly patients (mean age of 86.2 years for severe cases vs. 53.6 years for moderate cases) (Supplementary Fig. 1a and Supplementary Table 1). Finally, we cannot exclude the possibility that comorbidities not considered here may have certain influence on the overall results.

On average, 77.2% of the reads aligned to the human genome. (Supplementary Table 2). A minimal proportion (0.0001%) of the small RNAs recovered from infected patients matched the SARS-CoV-2 genome and only eight of them (Supplementary Table 3) were coincident with previously reported virus-derived sRNAs sequences^27^. 70% of the total clean reads were annotated as host-derived elements, 6.01% of the sequences were identified as microorganism-derived elements and the remaining reads (24.88%) were considered as unidentified. Figure 1d shows the distribution of length of the identified reads. Only reads with a length between 12 and 34 nt were subsequently analyzed. Considering the overall profile, those of 21-23 nt were predominant in both infected-patient and control samples. However, a remarkable peak of reads of 32-33 nt was also observed in the libraries obtained from the infected patients.

### SARS-CoV-2 infection leads to a global alteration of the sncRNA landscape

**Fig. 2:**
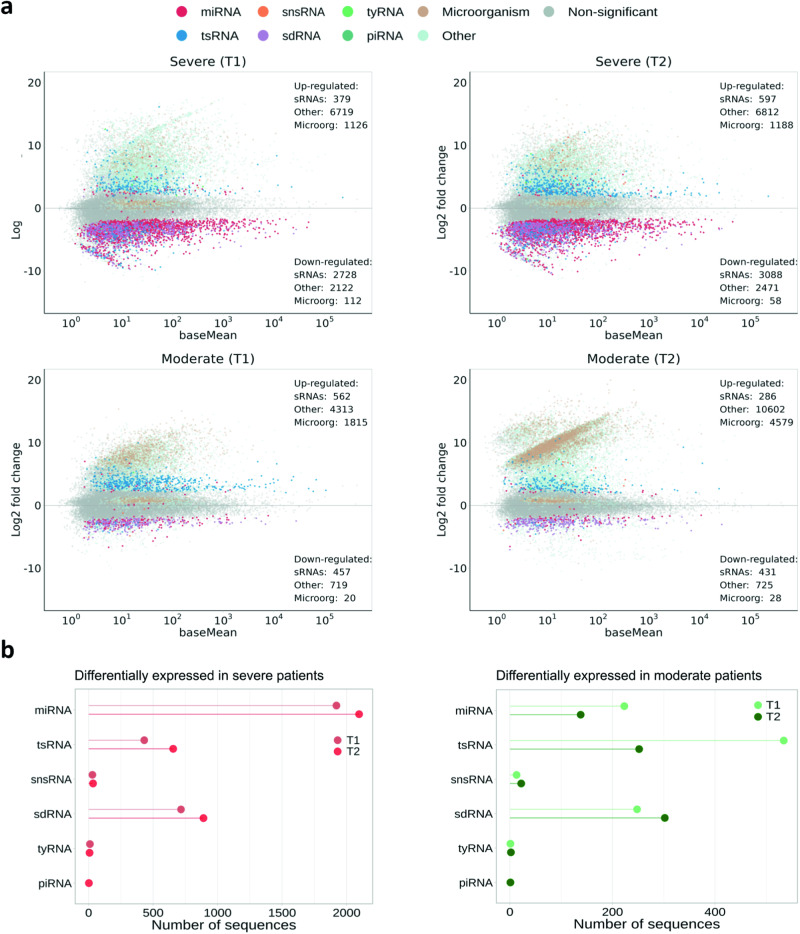
Global landscape of altered sRNAs upon SARS-CoV-2 infection. **a** Graphic representation of the expression values (DESeq2) of sRNA sequences for the different conditions against control samples. Each dot corresponds to a given sRNA expression value. Colors indicate significant differential expression with |log2*FC* | > 0.585 and *FDR* < 0.05 for the different sRNA families (sRNAs with non-significant differential expression are in gray). The category Other is used to label those endogenous sRNAs that could not be annotated or were annotated as miRNAs with a length not compressed between 19 and 24 nt, lncRNAs, rRNAs, scaRNAs, protein-coding or miscellaneous RNAs. sRNAs annotated as derived from microorganisms are colored in light brown. **b** Detail of the number of sequences differentially expressed for each sRNA family in the four conditions analyzed. miRNA micro RNA, tsRNA tRNA-derived small RNA, snsRNA small nuclear-derived RNA, sdRNA small nucleolar-derived RNAs, tyRNA tiny RNA and piRNA: Piwi-interacting RNA.

The effect of SARS-CoV-2 on the accumulation of host sncRNAs was assessed by pairwise comparisons between control and infected samples. Only the sequences with a sufficient fold change upon infection (i.e., matching the condition log2*FC* > 0.585, corresponding to *FC* > 1.5, or log2*FC* < -0.585, corresponding to *FC* < 0.667, with *FDR* < 0.05) were considered as significantly differentially expressed (Fig. 2a).

Altered sequences were categorized according to their homology with the most relevant classes of regulatory sncRNAs (miRNAs, tsRNAs, piRNAs, tyRNAs, sdRNAs, and snsRNAs) (Supplementary Table 4). The remaining host-derived sncRNAs with differential expression (categorized as Other) and the exogenous sRNAs identified as derived from genomes of diverse microorganisms were not included for subsequent analyses, focusing here on endogenous sncRNAs. A total of 3107 and 3685 unique sncRNAs were differentially expressed in response to severe infection at T1 and T2, respectively. However, the reactive unique sncRNAs recovered from patients with moderate symptoms were 1019 at T1 and 717 at T2 (Supplementary Table 4). In general, miRNAs, sdRNAs, and tsRNAs were altered in response to SARS-CoV-2 in all analyzed samples (Fig. 2b and Supplementary Table 4). Of note, miRNAs were predominantly affected in the case of severe disease at both T1 (1921 unique sequences, 61.8%) and T2 (2097 unique sequences, 56.9%). In contrast, tsRNAs were the most commonly altered sncRNAs in patients with moderate disease at T1 (534 unique sequences, 52.4%), while sdRNAs were at T2 (302 unique sequences, 42.1%). A minor impact of SARS-CoV-2 on the expression of tyRNAs, snsRNAs, and piRNAs was observed (Supplementary Table 4).

To further assess the behavior of the altered sncRNAs upon infection, we compared the temporal evolution (at T1 and T2) of the accumulation of differentially expressed miRNAs, sdRNAs, tsRNAs, and tyRNAs [estimated by reads per million (RPM)]. Data shown in Supplementary Fig. 2 indicate that, although miRNAs were predominantly down-regulated in response to severe and moderate infection at both T1 and T2, a severe infection leads to a greater impact over time (*i.e*., more alterations at T2 than at T1). In general, all miRNA sequences pertaining to the same family showed a comparable accumulation trend in response to SARS-CoV-2 (Supplementary Figure 3). Regarding tsRNAs, up-regulation was the general response to infection (Supplementary Figure 2). While the intensity of the relative accumulation was comparable at T1 and T2 in the case of severe infection, this intensity notably decreased at T2 with respect to T1 when the outcome of infection was moderate. Finally, differentially expressed tyRNAs were more abundant in patients exhibiting severe disease at both T1 and T2 than in patients with moderate symptoms.

### miRNA expression is predominantly down-regulated in response to SARS-CoV-2 infection

A total of 580 high-confidence unique sequences belonging to 143 known miRNA families were identified as significantly differentially expressed in infected patients with severe and moderate symptoms (Supplementary Figure 3 and Supplementary Table 5). The miRNA response was more evident in patients with severe disease, with 110 and 118 altered miRNA families identified at T1 and T2, respectively. In contrast, only 33 miRNA families at T1 and 17 at T2 were identified as differentially expressed when only moderate symptoms appear. Down-regulation of miRNAs was the general response to infection. A high proportion of miRNA families responding to infection were consistently down-regulated in patients with severe symptoms (90.0% at T1 and 94.0% at T2). In the case of moderate symptoms, down-regulated families accounted for 60.6% (at T1) and 64.7% (at T2) of differentially expressed miRNAs. Only four miRNA families (hsa-miR-10, hsa-miR-27, hsa-miR-122, and hsa-miR-203) showed an inconsistent response, their corresponding elements being ambivalently up- or down-regulated in the four conditions analyzed. In 79 perturbed miRNA families, differential expression was associated with the infection level (75 to severe and 4 to moderate) and only two were time-dependent (hsa-miR-576 at T1 and hsa-miR-129 at T2) (Supplementary Table 5).

**Fig. 3:**
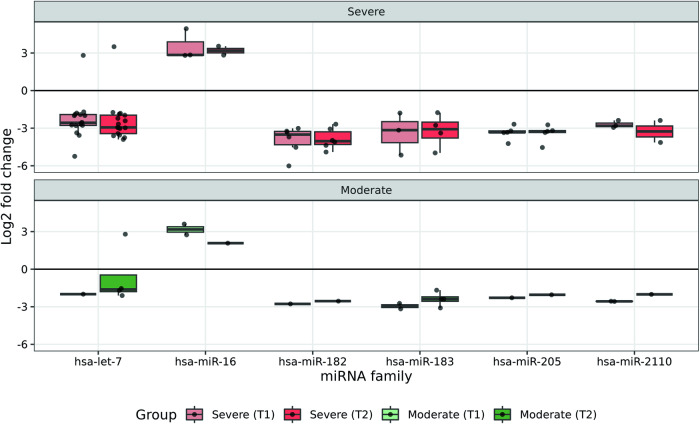
Expression levels of different miRNA families with consistent differential expression in the four groups analyzed. Boxplot representation (on the top, patients with severe symptoms; on the bottom, patients with moderate symptoms). Each dot represents the expression value (log2*FC*) of a sequence in infected patients (T1 or T2) with respect to the non-infected control. In boxplots, the central lines depict the median, while the box boundaries represent the upper and lower quartiles. The whiskers extend to the first or last data point within 1.5x the interquartile range of the box boundaries in the lower and upper directions, respectively.

Interestingly, six miRNA families displayed a consistent response in the four groups of infected patients (Fig. 3 and Supplementary Table 5). Five of these miRNA families (let-7, hsa-miR-182, hsa-miR-183, hsa-miR-205, and hsa-miR-2110) were down-regulated and only one (hsa-miR-16) was up-regulated in response to SARS-CoV-2 infection. These results are in agreement with previous reports that have largely described down-regulation of hsa-let-7 in COVID-19 patients^28^. In addition, significant down-regulation of hsa-miR-183 and hsa-miR-205 and up-regulation of hsa-miR-16 have been found in blood also in COVID-19 patients^29,30^. The remaining down-regulated miRNA families (hsa-miR-182 and hsa-miR-2110) have not been previously identified as differentially expressed upon SARS-CoV-2 infection^28^. To obtain a comprehensive understanding of the endogenous pathways susceptible to be influenced by the alterations observed in miRNA population, we analyzed 212 experimentally validated targets (according to the conditions established in the Methods section) of the sequences included in the six families of miRNAs with consistently differential expression in response to SARS-CoV-2 infection (Supplementary Table 6). Kyoto Encyclopedia of Genes and Genomes (KEGG) pathway analysis revealed miRNA-target genes significantly enriched in diverse regulatory pathways (Supplementary Fig. 4). Considering the top ten enriched pathways, the phosphatidylinositol 3-kinase/protein kinase B/mammalian target of rapamycin (PI3K/Akt) signaling and the EGFR tyrosine kinase inhibitor resistance, two pathways associated to SARS-CoV-2 infection^31,32^, were predominantly enriched in both Severe and Moderate cases. In contrast, miRNA targets enriched in the age-associated receptor for advanced glycation endproducts (AGE-RAGE) signaling, a pathway associated to COVID-19 severity^33^, were only observed in patients requiring intensive care (severe cases).

### 5’ tRNA halves derived from Glu- and Gly-tRNAs encompass the major tsRNA alteration upon SARS-CoV-2 infection

Differentially expressed tsRNAs were classified according to their tRNA precursor (Supplementary Table 7). As shown in Supplementary Figure 5, altered tsRNAs derived from a wide range of tRNAs were detected in infected patients. Remarkably, tsRNAs derived from glutamyl- (Glu-) and glycyl-tRNAs (Gly-tRNAs) were the predominant in the list (Supplementary Fig. 6). Using the median number of total differentially expressed reads as a proxy of relative accumulation, on the one hand, we obtained 4.8·10^4^ RPM at T1 and more than 5·10^4^ at T2 for Glu-tsRNAs when the symptoms are severe. In the case of a moderate disease, the accumulation of Glu-tsRNAs dropped, especially at late times where only one sequence derived from this tsRNA is differentially expressed (*i.e*., about 3·10^4^ RPM at T1 and 0.19 RPM at T2). On the other hand, we obtained a poor relative accumulation of Gly-tsRNAs when the symptoms are severe (*i.e*., about 2.5·10^2^ RPM at T1 and 2.7·10^3^ RPM at T2). In contrast, Gly-tsRNAs accumulated substantially in patients with moderate symptoms at early times (*i.e*., about 5·10^4^ RPM at T1 and 5.9·10^3^ at T2).

**Fig. 4:**
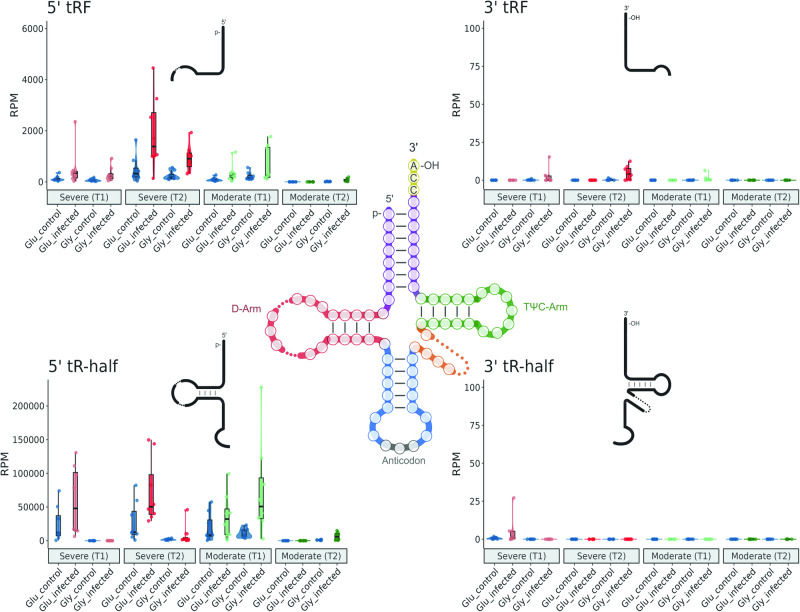
Expression profiles of altered Glu-tRNA- and Gly-tRNA-derived sRNAs for each condition. Four classes of tRNA-derived sRNAs are represented: 5’tRF, 3’tRF, 5’tR-half, and 3’tR-half. Dots indicate the absolute accumulation in reads per million (RPM) of differentially expressed sequences classified as Glu-tsRNA or Gly-tsRNA in control and infected samples for each condition (severe/moderate symptoms, T1/T2 time). In boxplots, the central lines depict the median, while the box boundaries represent the upper and lower quartiles. The whiskers extend to the first or last data point within 1.5x the interquartile range of the box boundaries in the lower and upper directions, respectively.

We next inspected the particular type of tsRNA that accumulates in response to the virus. We classified these highly accumulating tsRNAs according to their biogenesis as if they were derived from 5’ or 3’ arms and according to their length (*i.e*., halves for ≥ 30 nt or tRF for < 30 nt). Of note, 5’ tsRNA-halves were the more abundant species, whereas tsRNAs of smaller size (5’ tRFs) were less abundant (Fig. 4). A marginal proportion of tsRNAs derived from the 3’ arm of both Glu- and Gly-tRNAs was recovered. Collectively, these results suggest that these tsRNAs might be exploited as biomarkers, not only to infer the presence of the virus (through the monitoring of Glu-tsRNAs), but also to predict the outcome of infection (elevated levels of Gly-tsRNAs at early times were only observed in patients with moderate symptomatology).

### SARS-CoV-2 infection leads to a down-regulation of C/D-box sdRNAs

Small RNAs derived from 77 parental snoRNAs showing differential expression were identified. These sdRNAs were predominantly characterized by significant down-regulation in all four groups of COVID-19 patients analyzed in this work (Supplementary Figure 7 and Supplementary Table 8). This decrease was more evident in patients with severe symptomatology. In this case, down-regulated sdRNAs derived from 60 (at T1) and 63 (at T2) different parental snoRNAs (Supplementary Figure 7). Although down-regulation was also the predominant pattern in patients with moderate symptoms, fewer classes of sdRNAs were observed (32 at T1 and 26 at T2). Regarding the type of parental snoRNAs, a high proportion of them were categorized as of C/D-box type. In tune with previous characterizations, those sequences close to 27 nt in length were the predominant class of sdRNAs recovered (Supplementary Figure 8). Interestingly, we found 23 different precursors associated systematically with the four infection conditions analyzed (Supplementary Table 8).

### SARS-CoV-2 infection alters the accumulation of tyRNAs

According to their well-established biogenesis pathway, only 13–14 nt in length sequences fully matching the 5’ end of a mature miRNA were considered high-confidence miRNA-derived tyRNAs^18^. We detected 8 tyRNAs with significant differential accumulation in COVID-19 patients (Supplementary Figure 9 and Supplementary Table 9). As occurred with the miRNA population, tyRNA alteration was more evident in patients with severe symptoms. In this case, three tyRNAs were significantly down-regulated at T1 (hsa-miR-378-tyRNA, hsa-miR-149-tyRNA, and hsa-miR-200-tyRNA) and other three up-regulated (hsa-miR-5087-tyRNA, hsa-miR-125-tyRNA, and hsa-let-7-tyRNA). Moreover, two tyRNAs were significantly up-regulated at T2 (hsa-miR-10400-tyRNA and hsa-miR-210-tyRNA), while hsa-miR149-tyRNA was also down-regulated at late times. With respect to patients with moderate symptoms, hsa-miR-5807-tyRNA was up-regulated at both T1 and T2.

### Sequencing data verification by stem loop RT-qPCR

To verify in part our RNA sequencing analyses by an independent experimental method, we used stem loop reverse transcription quantitative polymerase chain reaction (RT-qPCR) to process additional and independent nasopharyngeal samples derived from SARS-CoV-2-infected and non-infected patients. We selected five different sequences that resulted overexpressed upon SARS-CoV-2 infection. These sequences represented three different types of sRNAs: two miRNAs (hsa-miR-16-3p and hsa-miR-16-5p), two tsRNAs (Glu-tsRNA and Gly-tsRNA), and one sdRNA (snord-55). As it is shown in Supplementary Table 10, the sRNA accumulation values estimated by stem loop RT-qPCR for the five analyzed sequences were consistent with those obtained by RNA sequencing, also showing significant overexpression in response to SARS-CoV-2.

## Discussion

Different studies have described the impact of SARS-CoV-2 infection on the sncRNA population of humans. However, these efforts have been predominantly focused on the analysis of alterations in circulating miRNA profiles recovered from blood or plasma^28,34^. Here, we performed an integrative analysis aimed at assessing the impact of infection on the global landscape of the different regulatory RNAs in human cells. For that, nasopharyngeal samples obtained from COVID-19 patients with different disease severity and at two time points were processed. Our results suggest that SARS-CoV-2 infection is associated with a significant imbalance of diverse sdRNAs, miRNAs, tsRNAs, and tyRNAs.

The detection of diverse miRNA families with significant differential expression in COVID-19 patients coincides with early studies showing that SARS-CoV-2 infection provokes a robust host miRNA response that might improve COVID-19 detection and patient management^35^. A first global analysis of the miRNA response to infection showed a strong association between disease severity and alterations in the miRNA population. The number of differentially expressed miRNAs was higher in patients requiring intensive care unit admission (severe symptoms) than in those exhibiting moderate symptoms. This observation associating disease intensity with alterations in miRNA expression is consistent with previous findings from the analysis of sRNAs recovered from blood^8^ and plasma^9^ samples of SARS-CoV-2-infected patients. Regarding miRNAs with differential accumulation in the four infection phases/stages analyzed here, it is important to highlight the presence of hsa-let-7. Members in the miRNA let-7 family are highly conserved across species, from *C. elegans* (the organism in which it was discovered) to humans^36^. In mammals, hsa-let-7 is involved in the regulation of critical physiological processes, such as organ development, growth, tissue regeneration, metabolism, cancer proliferation and modulation of viral infection^36,37^. A recent work has reported that a member of this family (hsa-let-7b) is involved in the regulation of *Angiotensin-converting enzyme 2* (ACE2) and *Dipeptidyl peptidase-4* (DPP4), two primary receptor proposed to play a key and complementary role in virus entry and disease progression^38^. Additionally, computational predictions have suggested that hsa-let-7 might target SARS-CoV-2^39^ and might also participate in the regulation of target genes involved in immune response pathways^40^. Our finding that the hsa-let-7 family is highly down-regulated in COVID-19 patients at different stages of infection (early and late) and disease intensity (moderate and severe) provides further support to the relationship between hsa-let-7 and SARS-CoV-2. The hsa-miR-182 was another down-regulated miRNA strongly associated with the infection and, to our knowledge, this miRNA family had not previously been reported as perturbed in COVID-19 patients. This particular observation may be due to the fact that, in general, previous infection-associated miRNA analyses have been performed using blood or plasma, rather than nasopharyngeal samples. Interestingly, it has been hypothesized through computational studies that the hsa-miR-182 miRNA family might recognize the SARS-CoV-2 genome as a potential target and, consequently, that its reduced expression might be associated with host susceptibility to infection^41^. Regarding the KEGG pathways enriched for validated miRNA-targets, at least three of them PI3K/Akt^42^, EGFR^43^ and RAGE^44^ have been previously suggested as potential pharmacological targets for the treatment of COVID19.

An emerging question related to COVID-19 is how can the virus infection lead to the alterations observed in endogenous miRNA population. The direct interaction between the virus and human proteins could be one of the causes responsible of the transcriptional effects associated with diseases induced by coronavirus in general^45^ and SARS-CoV-2 in particular^46,47^. Interestingly, it has been showed that the SARS-CoV-2 N protein is able to bind to DICER1 and that the GO term “Regulation of gene silencing mediated by miRNAs” is significantly enriched when the global SARS-CoV-2-human protein-protein interactome is analyzed^47,48^, suggesting the potential capacity of certain viral proteins to interfere in the miRNA biogenesis. This possibility is consistent with the previously reported the functional interaction between the Human-Nipah virus M protein and the DICER1-TARBP2 complex^49^. Moreover, the observation that the expression of genes related to miRNA processing is not affected during SARS-CoV-2 infection^50^ provides additional support to this notion.

Recent studies have reported the differential expression of tsRNAs in blood^8^, plasma^9^, and nasopharyngeal swabs^51,52^ from COVID-19 patients. However, the small number of samples or the lack of information about the severity and time of infection limit these initial analyses. This point acquires particular relevance considering that altered tsRNA profiles in diverse cell type or tissues emerge as great tools from which to disclose novel potential biomarkers for early diagnosis and prognosis of physiological disorders^53^ such as diverse cancer types^54,55^, neuronal disorders^56^ and viral infections^57,58^. Our results reinforce the significant expression alteration of tsRNAs in response to SARS-CoV-2 infection. Sequences derived from Glu- and Gly-tRNA precursors were the most abundant in COVID-19 patients. In particular, the Glu-tsRNAs were the predominant forms in severe infections at both time points, whereas the accumulation of Gly-tsRNAs was increased in moderate cases at early stages of the infection. Considering the type of tsRNAs, we observed that 5’ variants accounted for virtually all Glu- and Gly-tsRNAs recovered from infected patients. In contrast, 3’ variants of tsRNAs were recently described as highly accumulated markers in blood samples of patients with severe symptoms^8^. These results suggest that, although differential accumulation of tsRNAs emerges as a general phenomenon associated with SARS-Cov-2 infection, its specific processing (and consequently the class of tsRNA detected) could be related to the disease intensity, infection stage, and the type of sample considered for the analysis. In this regard, a recent study conducted in cell culture has found that a group of tsRNAs upregulated in response to SARS-CoV-2 infection was not altered during infection with SARS-CoV or other viruses tested^59^. Even though the role played by tsRNAs during SARS-CoV-2 infection is unknown, it is important to note that it has been proposed that certain tRNA-derived RNAs acting as siRNAs are able to silence in *trans* host target transcripts in order to favor the replication of respiratory syncytial virus (RSV)^57,60^. Further studies are needed to elucidate whether a similar tsRNA-mediated mechanism underlies SARS-CoV-2 pathogenesis.

While the functional relevance of sdRNAs is currently unknown, their abundance suggests yet poorly described regulatory roles, such as a novel source of miRNAs and siRNAs^61^. According to our results, various sdRNAs were differentially expressed during SARS-CoV-2 infection. Although this response was more evident in patients with severe disease, a significant down-regulation of sequences derived from various parental snoRNAs was observed in all four cases analyzed. In agreement with what has been described in other pathological conditions, such as cancer^62^ or further viral infections^63^, sdRNAs derived from snoRNA C/D-box precursors were the predominant forms differentially expressed in COVID-19 patients. With the exception of one study based on microarray technology^25^, our RNA-Seq results are pioneer in supporting a link between SARS-CoV-2 and the sdRNA population.

Another contribution of our research is the identification of differential tyRNAs in COVID-19 patients. TyRNAs constitute an emerging and intriguing class of sncRNAs, and they had not previously been associated with SARS-CoV-2 infection. In contrast to the observation for the miRNAs (their well-established precursors), up-regulation of tyRNAs was the predominant response to infection. To date, it is not widely accepted that tyRNAs constitute a representative class of regulatory sRNAs in mammals, and the only functional role proposed for these sRNAs is associated with neurodegenerative diseases^18^, making it difficult to draw any conclusion about their relationship to SARS-CoV-2 infection. In this regard, the potential functional role of this little-known regulatory layer during disease would require further attention.

In sum, our results provide an integrative view of the sncRNA signature in severe and moderate cases of COVID-19 at different time points of infection. We acknowledge that the lack of subsequent functional validation of the regulatory activity of the identified sncRNAs and the fact that the study was conducted using nasopharyngeal samples, rather than other biological fluids that could offer a more precise picture of circulating sncRNAs (such as blood or lymph), might constitute a limitation of our study. However, the valuable information derived from our detailed computational analysis could be used for the identification of alternative biomarkers for diagnosis and/or prognosis. Indeed, it has recently been proposed that accurate expression profiling of disease-associated miRNAs (MDAs) combined with machine learning or deep learning strategies can serve as an innovative diagnostic tool for various physiological disorders in patients^64,65^, including certain types of sclerosis^66^, Lyssavirus infection^67^, and COVID-19^68^. Hence, this comprehensive study might provide, once the regulatory pathways involved in the sncRNA-mediated response are characterized, a new perspective for the development of innovative therapeutic and/or prophylactic strategies for the control and management of SARS-CoV-2 infection.

## Methods

### Patient samples

Nasopharyngeal (swabs or aspirates) samples corresponding to infected and non-infected patients with SARS-CoV-2 were obtained from the Clinic University Hospital of Valencia (Spain). Samples were collected in a universal transport medium (Beckton Dickinson or Copan Diagnostics). Data were anonymized, so that the patient’s identification data were separated from the clinical-care and demographic data. In any case, a waiver of informed consent was requested to the ethical committee to perform this research study. The ethical committee of the Clinic University Hospital of Valencia approved this study (order #2020/221). Samples were gathered during the first wave of the pandemic (April–June 2020), when only ancestral SARS-CoV-2 variants were in circulation^69^. In total, 50 samples were used in this study, which included 20 SARS-CoV-2 positive samples, collected at two different times (separated by 14 ± 4 days), and ten non-infected control samples. COVID-19 patients were classified according to whether they showed severe (10 cases) or moderate (10 cases) symptoms. The severe category comprises patients who required admission to the intensive care unit.

### RT-qPCR

Nucleic acid extraction was performed from undiluted samples using the Qiagen EZ1 Viral extraction kit or the DSP virus Pathogen Minikit using EZ1 or Qiasymphony Robot instruments (Qiagen), respectively. The following commercially available RT-qPCR assay for SARS-CoV-2 testing was used in the hospital: REALQUALITY RQ-2019-nCoV from AB ANALITICA on the Applied Biosystems 7500 instrument. This RT-qPCR assay analyses the E (envelope) and RdRp (RNA-dependent RNA polymerase) genes of SARS-CoV-2 in a single reaction^70^.

### Total RNA isolation

Samples were inactivated by heat shock (30 min at 60 °C) before proceeding. Samples were split in two fractions of 250 μL and each fraction was subject to the following protocol. To 250 μL of sample, 750 μL of TRIzol LS reagent (Invitrogen) was added and mixed to homogenize. Samples were incubated for 5 min at room temperature and the mix was transferred to phase-lock gel tubes (Invitrogen) and then were incubated for additional 3 min. At this point, 200 μL of chloroform-isoamyl alcohol was added to each sample and then thoroughly mixed by shaking. Samples were incubated for 15 min at room temperature and centrifuged for 15 min at 12,000 g and 4 °C. 500 μL of the aqueous phase of each sample was transferred to a new tube and 500 μL of isopropanol was added. Samples were incubated for 10 min at 4 °C and then centrifuged for 10 min at 12000 g and 4 °C. Supernatants were discarded and RNA pellets were washed twice with 750 μL 75% ethanol (5 min, 7500 g, 4 °C). Supernatants were discarded and RNA pellets were left air dry for 5 min. Then, RNA pellets were resuspended in 25 μL of DEPC water and 1 μL of each sample was quantified using a NanoDrop (Thermo) device.

### Small RNA sequencing

Production and sequencing of the libraries were carried out by Novogene (https://en.novogene.com) according to their standard procedures. Briefly, sRNA libraries were directly generated from total RNA using TruSeq Small RNA Library Prep Kit (Illumina). The 3’ and 5’ adaptors were sequentially ligated to the RNA prior to reverse transcription and cDNA generation. cDNAs were enriched by PCR to create the indexed double stranded cDNA library. Size selection was performed using 6% polyacrylamide gel. The quantity of the libraries was determined by quantitative real-time PCR and an equimolar pooling of the libraries was performed. The cDNA libraries were sequenced following a single-batch strategy on a NOVASEQ 6000 machine (Illumina).

### Quality control and quantification of the sequences

The adaptor removal and size selection of the raw FASTQ files was performed with Unitas (v1.8.0)^71^ selecting the sequences in the range of 12–34 nt. After visual inspection of Unitas summary, those files whose overall profile might denote sample degradation, high percentage of unannotated sequences, and/or high percentage of sequences annotated as rRNA, protein coding, or lncRNAs were excluded. After this filtering, two samples per patient group were discarded for subsequent analysis.

For quality control purposes, the remaining libraries were aligned against the human genome of GENCODE (47) GRCh38 (v.38), those sequences that did not align against the human genome were then aligned with the SARS-CoV-2 genome (NC_045512.2). The alignment was performed using Bowtie (v1.3.1)^72^ with the parameters -n 1 -l 10 -k 1 –best. Quantification and removal of sequences containing indeterminacies were performed using in-house Python scripts. Both absolute and normalized (RPM) counts were obtained for each sample. The individual results were joined to create the matrix of counts used for differential expression analysis and visualization purposes. To study the correlation exhibited by the sRNA expression profiles among the different groups and samples, principal component analysis (PCA) was used. PCA was performed using the prcomp function of the stats R package (v4.2.2) (https://www.r-project.org/) with the matrix of absolute counts (filtered considering only the sequences with 5 counts in at least five samples of any of the groups), which was normalized by size factor and transformed with the vst function, both functions of the DESeq2 R package (v1.36)^73^. The 1000 sequences with the highest variance were selected for plotting.

### Annotation of the sequences

The annotation of the clean reads of each of the libraries was performed with Unitas, which internally uses the aligner Seqmap (v1.0.13)^74^, with the following parameters: allowing one internal modification (default value) but 0 non-template 3’ nucleotides (-tail 0) for miRNA annotation, and allowing one mismatch (default value) and one indel (-insdel 1) for the rest of ncRNA annotation. The software for the annotation used the following resources: miRBase (v22.1)^75^, gtRNAdb (accessed on May 2023)^76^, piRNA cluster database (accessed on May 2023)^77^, Ensembl (release 108), EnsemblGenomes (release 55)^78^ and SILVA (release 132)^79^. All the individual annotations obtained per sample were filtered, selecting only the first annotation provided by the software, and collapsed into one unified annotation at unique sequence level. For the study of miRNAs certain modifications were made on the Unitas classification. For the annotation of differentially expressed miRNAs, only those sequences annotated as such and between 20 and 24 nt were considered “true miRNAs”, the rest were classified as “miRNA-like”. In addition, for the analysis by families, only those “true miRNAs” annotated with 0 mismatches were considered. As for tyRNAs annotation, we selected those “miRNA-like” sequences of 13 and 14 nt, mapped with Scram (v0.2.2)^80^ against miRBase mature miRNA sequences (v22.1) and selected those sequences that mapped on the positive strand and with start position 1 or 2. Regarding the analysis of tsRNAs, Unitas results were filtered to discard sequences coming from mitochondrial tRNAs, and only tsRNAs from the categories 5’ tRF, 3’ tRF, 5’ tR-half and 3’ tR-half were considered. Exogenous small RNA identification of the reads that could not be annotated by Unitas was performed with Centrifuge (v1.0.4)^81^ against the database provided by the software authors (hpvc) containing the human genome, prokaryotic genomes and viral genomes downloaded from Genbank on March 2020 (https://zenodo.org/record/3732127/files/h+p+v+c.tar.gz?download=1).

### Differential expression analysis

DESeq2 was utilized to conduct a differential expression analysis, which uses raw read counts as input and estimates the Log2 Fold Change (log2*FC*) along with its corresponding standard error (SE). The raw read counts matrix was filtered considering only the sequences with 5 counts in at least five samples of any of the groups. Raw counts were normalized with the DESeq2 median of ratios method. Hypothesis testing was performed by a Wald test looking for log2*FC* > 0.585 (*i.e*., FC > 1.5 or FC < 0.667), and the Benjamini-Hochberg procedure was subsequently used to adjust for multiple hypothesis testing. To be considered a differentially expressed sequence an *FDR* threshold of < 0.05 was applied. The LFC shrinkage function in DESeq2, lfcShrink(), was used to shrink fold changes for sequences with higher variance.

### miRNA target identification and KEGG enrichment analysis

The miRNA host targets were retrieved from miRTarBase^82^. The human miRNA target interaction (MTI) file (release 9.0) was filtered to keep only those MTIs supported by at least two validation methods targeted as “*strong evidence*”. The members of the six miRNA-families consistently differentially expressed in the four groups of analyzed infected patients were intersected with the targets. KEGG^83^ enrichment analysis of the resultant miRNA-target genes was performed using the function enrichKEGG of the package ClusterProfiler^84^ (v4.10).

### Stem loop RT-qPCR

Quantification of five selected sRNAs was performed from nasopharyngeal samples corresponding to 3 non-infected and 3 SARS-CoV-2 infected patients (Supplementary Table 10), starting from small RNA ( < 200 nt) enriched fractions using REALTOTAL microRNA Kit (RBMER14, Durviz) according to the manufacturer’s instructions. Stem-loop-specific reverse transcription for miRNAs detection was performed as previously described in ref. ^85^ using a RevertAid cDNA Synthesis Kit (Thermo Scientific). All analyses were done in triplicate on a QuantStudio qPCR instrument (Thermo Scientific™) using a standard protocol. Relative RNA expression was quantified by the comparative ΔΔ*C*_*T*_ method^86^ and normalized to the geometric mean of the small-nucleolar RNAs RNU48 (AN X96648.1), a reference gene commonly used for miRNAs estimation by RT-qPCR in humans^87^. The statistical significance of the observed differences was evaluated by the paired *t*-test. Primers used for amplification assays are detailed in the Supplementary Table 11.

### Reporting summary

Further information on research design is available in the Nature Research Reporting Summary linked to this article.

### Supplementary information

Reporting summary

Supplementary Material

Supplementary Table 1

Supplementary Table 2

Supplementary Table 3

Supplementary Table 4

Supplementary Table 5

Supplementary Table 6

Supplementary Table 7

Supplementary Table 8

Supplementary Table 9

Supplementary Table 10

Supplementary Table 11

## Data Availability

The data have been deposited with links to BioProject accession number PRJNA982620 in the NCBI BioProject database (https://www.ncbi.nlm.nih.gov/bioproject).

## Code availability

All the necessary components, including scripts, software versions, and additional files required to replicate the study’s findings can be found on our GitHub page (https://github.com/ncRNA-lab/sRNA_covid19).
