# Supplementary material for: SARS-CoV-2 remodels the landscape of small non-coding RNAs with infection time and symptom severity
Source: NPJ Syst Biol Appl. 2024 Apr 17;10:41. doi: 10.1038/s41540-024-00367-z (PMC11024147; doi:10.1038/s41540-024-00367-z)
Supplement: Supplementary file 1 — Reporting summary [file 41540_2024_367_MOESM1_ESM.pdf]

nature portfolio

Corresponding author(s): Gustavo Gomez

Last updated by author(s): 2024/03/28

Reporting Summary

Nature Portfolio wishes to improve the reproducibility of the work that we publish. This form provides structure for consistency and transparency in reporting. For further information on Nature Portfolio policies, see our Editorial Policies and the Editorial Policy Checklist.

Statistics

For all statistical analyses, confirm that the following items are present in the figure legend, table legend, main text, or Methods section.

n/a	Confirmed	
☐	☒	The exact sample size (*n*) for each experimental group/condition, given as a discrete number and unit of measurement
☐	☒	A statement on whether measurements were taken from distinct samples or whether the same sample was measured repeatedly
☐	☒	The statistical test(s) used AND whether they are one- or two-sided<br>*Only common tests should be described solely by name; describe more complex techniques in the Methods section.*
☐	☒	A description of all covariates tested
☐	☒	A description of any assumptions or corrections, such as tests of normality and adjustment for multiple comparisons
☐	☒	A full description of the statistical parameters including central tendency (e.g. means) or other basic estimates (e.g. regression coefficient) AND variation (e.g. standard deviation) or associated estimates of uncertainty (e.g. confidence intervals)
☐	☒	For null hypothesis testing, the test statistic (e.g. *F*, *t*, *r*) with confidence intervals, effect sizes, degrees of freedom and *P* value noted<br>*Give P values as exact values whenever suitable.*
☒	☐	For Bayesian analysis, information on the choice of priors and Markov chain Monte Carlo settings
☒	☐	For hierarchical and complex designs, identification of the appropriate level for tests and full reporting of outcomes
☒	☐	Estimates of effect sizes (e.g. Cohen's *d*, Pearson's *r*), indicating how they were calculated

Our web collection on statistics for biologists contains articles on many of the points above.

Software and code

Policy information about availability of computer code

Data collection	No software was used for data collection.
Data analysis	Open source sotware used: R (v4.2.2), Python (>=3.8), Unitas (v1.8.0), Bowtie (v1.3.1), DESeq2 (v1.36), Seqmap (v1.0.13), Scram (v0.2.2), Centrifuge (v1.0.4), ClusterProfiler (v4.10).<br>Custom scripts required to replicate the study findings can be found on our GitHub page https://github.com/ncRNA-lab/sRNA_covid19.

For manuscripts utilizing custom algorithms or software that are central to the research but not yet described in published literature, software must be made available to editors and reviewers. We strongly encourage code deposition in a community repository (e.g. GitHub). See the Nature Portfolio guidelines for submitting code & software for further information.

Data

Policy information about availability of data

All manuscripts must include a data availability statement. This statement should provide the following information, where applicable:

- Accession codes, unique identifiers, or web links for publicly available datasets
- A description of any restrictions on data availability
- For clinical datasets or third party data, please ensure that the statement adheres to our policy

The data have been deposited with links to BioProject accession number PRJNA982620 in the NCBI BioProject database (https://www.ncbi.nlm.nih.gov/bioproject).

Research involving human participants, their data, or biological material

Policy information about studies with human participants or human data. See also policy information about sex, gender (identity/presentation), and sexual orientation and race, ethnicity and racism.

Reporting on sex and gender	In our study of 50 nasopharyngeal samples from SARS-CoV-2 patients, including 10 controls, 10 mild patients, and 10 severe patients, sampled at two time points, we collected anonymized data including age and self-reported sex. At the initial stages of analysis, we did not observe an association between sRNA expression in nasopharyngeal samples and sex (see Supplementary Figure 1), and therefore, sex-based analysis was not conducted.
Reporting on race, ethnicity, or other socially relevant groupings	In this study we have not used the constructs of race and/or ethnicity or any other socially constructed categorization.
Population characteristics	Patient demographics (age, sex an severity of COVID-19 symptoms) are provided in Supplementary Table 1.
Recruitment	In total, 50 samples were used in this study, which included 20 SARS-CoV-2 positive samples, collected at two different times (separated by 14 ± 4 days), and ten non-infected control samples. COVID-19 patients were classified according to whether they showed severe (10 cases) or moderate (10 cases) symptoms. The severe category comprises patients who required admission to the intensive care unit.
Ethics oversight	The ethical committee of the Clinic University Hospital of Valencia approved this study (order #2020/221).

Note that full information on the approval of the study protocol must also be provided in the manuscript.

Field-specific reporting

Please select the one below that is the best fit for your research. If you are not sure, read the appropriate sections before making your selection.

☒ Life sciences ☐ Behavioural & social sciences ☐ Ecological, evolutionary & environmental sciences

For a reference copy of the document with all sections, see nature.com/documents/nr-reporting-summary-flat.pdf

Life sciences study design

All studies must disclose on these points even when the disclosure is negative.

Sample size	In total, 50 samples were used in this study, which included 20 SARS-CoV-2 positive samples, collected at two different times (separated by 14 ± 4 days), and ten non-infected control samples. COVID-19 patients were classified according to whether they showed severe (10 cases) or moderate (10 cases) symptoms.
Data exclusions	After visual inspection of Unitas summary, those sRNA-seq libraries whose overall profile might denote sample degradation, high percentage of unannotated sequences, and/or high percentage of sequences annotated as rRNA, protein coding, or lncRNAs were excluded. After this filtering, two samples per patient group were discarded for subsequent analysis.
Replication	10 biological replicates per group.
Randomization	*Describe how samples/organisms/participants were allocated into experimental groups. If allocation was not random, describe how covariates were controlled OR if this is not relevant to your study, explain why.*
Blinding	Blinding was not required in this sudy as all biological replicates have been treated in the same way.

Reporting for specific materials, systems and methods

We require information from authors about some types of materials, experimental systems and methods used in many studies. Here, indicate whether each material, system or method listed is relevant to your study. If you are not sure if a list item applies to your research, read the appropriate section before selecting a response.

Materials & experimental systems

n/a	Involved in the study
☒	☐ Antibodies
☒	☐ Eukaryotic cell lines
☒	☐ Palaeontology and archaeology
☒	☐ Animals and other organisms
☒	☐ Clinical data
☒	☐ Dual use research of concern
☒	☐ Plants

Methods

n/a	Involved in the study
☒	☐ ChIP-seq
☒	☐ Flow cytometry
☒	☐ MRI-based neuroimaging

Plants

Seed stocks

Report on the source of all seed stocks or other plant material used. If applicable, state the seed stock centre and catalogue number. If plant specimens were collected from the field, describe the collection location, date and sampling procedures.

Novel plant genotypes

Describe the methods by which all novel plant genotypes were produced. This includes those generated by transgenic approaches, gene editing, chemical/radiation-based mutagenesis and hybridization. For transgenic lines, describe the transformation method, the number of independent lines analyzed and the generation upon which experiments were performed. For gene-edited lines, describe the editor used, the endogenous sequence targeted for editing, the targeting guide RNA sequence (if applicable) and how the editor was applied.

Authentication

Describe any authentication procedures for each seed stock used or novel genotype generated. Describe any experiments used to assess the effect of a mutation and, where applicable, how potential secondary effects (e.g. second site T-DNA insertions, mosiacism, off-target gene editing) were examined.
