# Supplementary material for: SARS-CoV-2 remodels the landscape of small non-coding RNAs with infection time and symptom severity
Source: NPJ Syst Biol Appl. 2024 Apr 17;10:41. doi: 10.1038/s41540-024-00367-z (PMC11024147; doi:10.1038/s41540-024-00367-z)
Supplement: Supplementary file 2 — Supplementary Material [file 41540_2024_367_MOESM2_ESM.pdf]

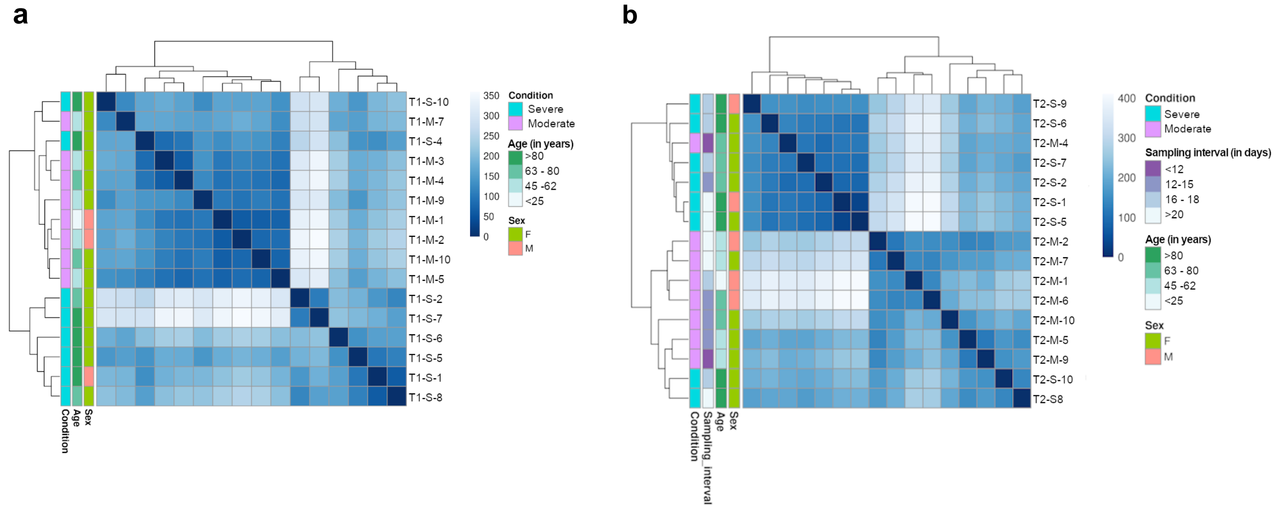

Supplementary Figure 1: Heatmap of sample-to-sample distances. A) Severe and moderate patients of the first sampling time point. B) Severe and moderate patients of the second sampling time point. Euclidian distance between samples was calculated using the dist() function from the stats R package on the matrix of absolute counts (filtered considering only the sequences with 5 counts in at least five samples of any of the groups and selecting 1000 sequences with higher variance). Pheatmap (v1.0.12) package was used to generate the heatmap.

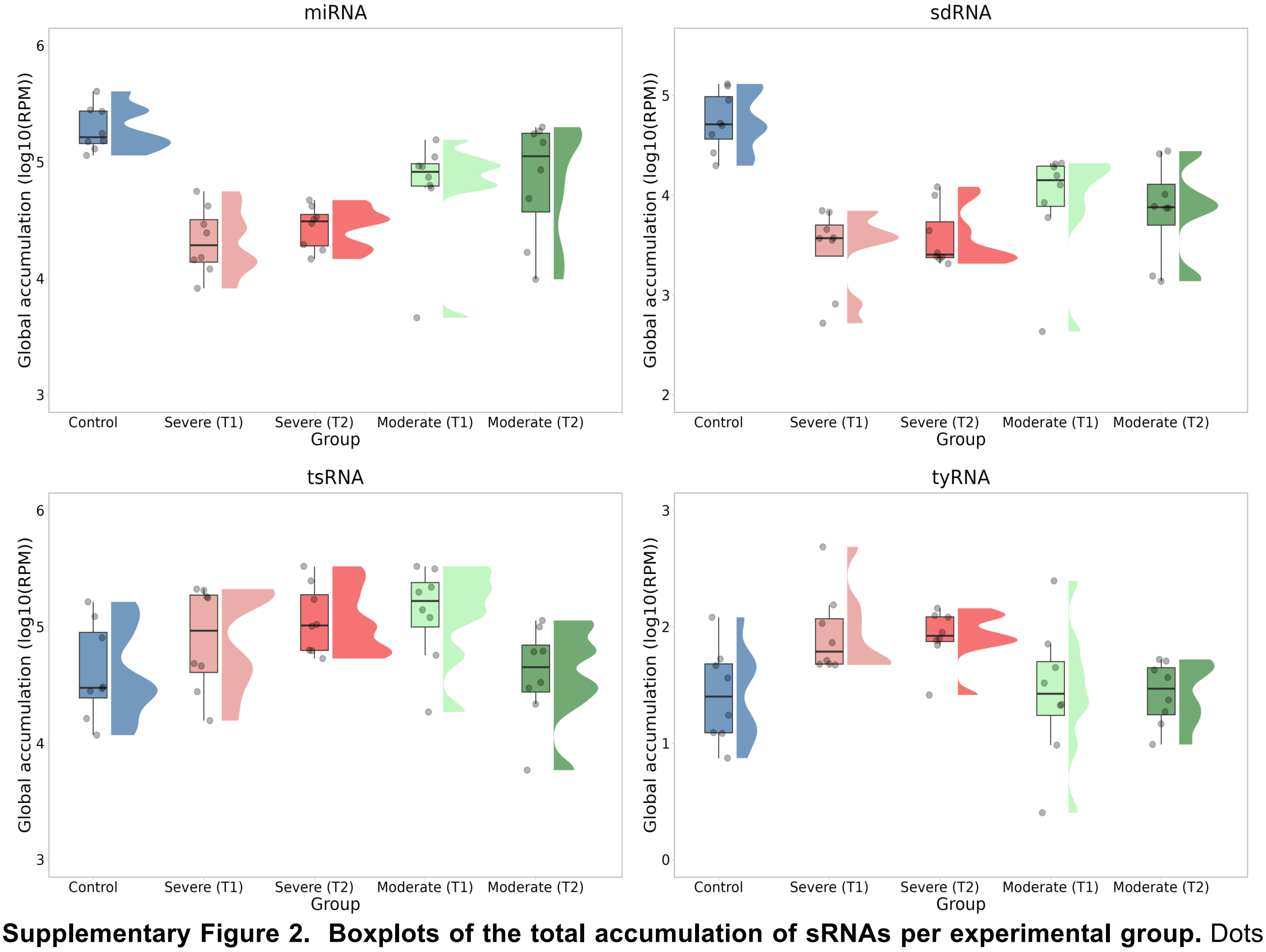

Supplementary Figure 2. Boxplots of the total accumulation of sRNAs per experimental group. Dots indicate the total accumulation of each sample. Only sRNAs that are differentially expressed in some of the contrasts are used for this representation. sRNAs classified as miRNAs, sdRNAs (small RNAs derived from snoRNAs), tsRNAs (tRNA-derived small RNAs), and tyRNAs (small RNAs derived from miRNAs) are represented. In boxplots, the central lines depict the median, while the box boundaries represent the upper and lower quartiles. The whiskers extend to the first or last data point within 1.5x the interquartile range of the box boundaries in the lower and upper directions, respectively. In boxplots, the central lines depict the median, while the box boundaries represent the upper and lower quartiles. The whiskers extend to the first or last data point within 1.5x the interquartile range of the box boundaries in the lower and upper directions, respectively.

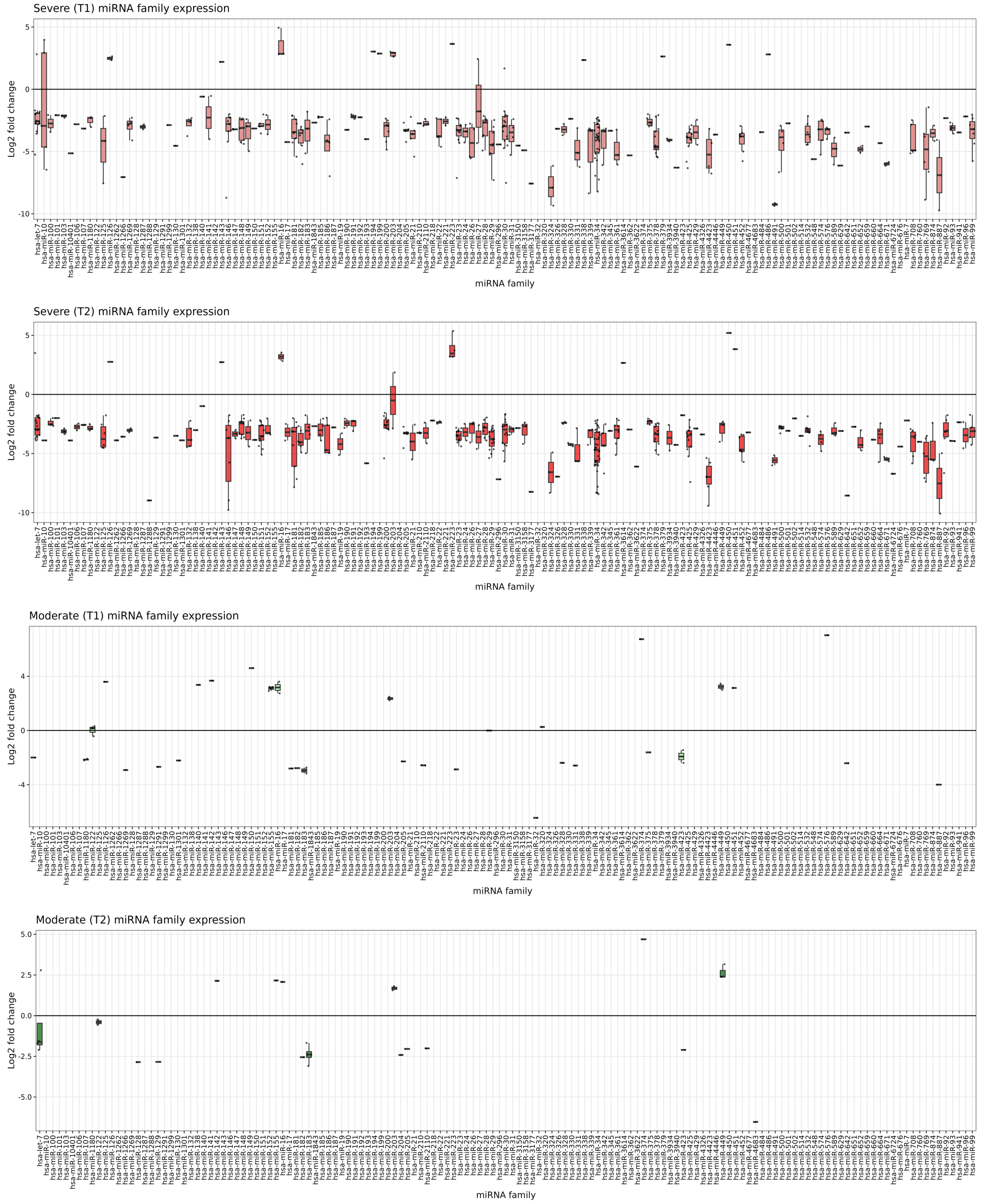

Supplementary Figure 3. Expression levels of miRNA families in patients with severe symptoms (T1 and T2) and patients with moderate symptoms (T1 and T2). Boxplot of the expression of each miRNA family. Each point represents the expression value (log2FC) of a sequence in infected patients versus non-infected controls. In boxplots, the central lines depict the median, while the box boundaries represent the upper and lower quartiles. The whiskers extend to the first or last data point within 1.5x the interquartile range of the box boundaries in the lower and upper directions, respectively. In boxplots, the central lines depict the median, while the box boundaries represent the upper and lower quartiles. The whiskers extend to the first or last data point within 1.5x the interquartile range of the box boundaries in the lower and upper directions, respectively.

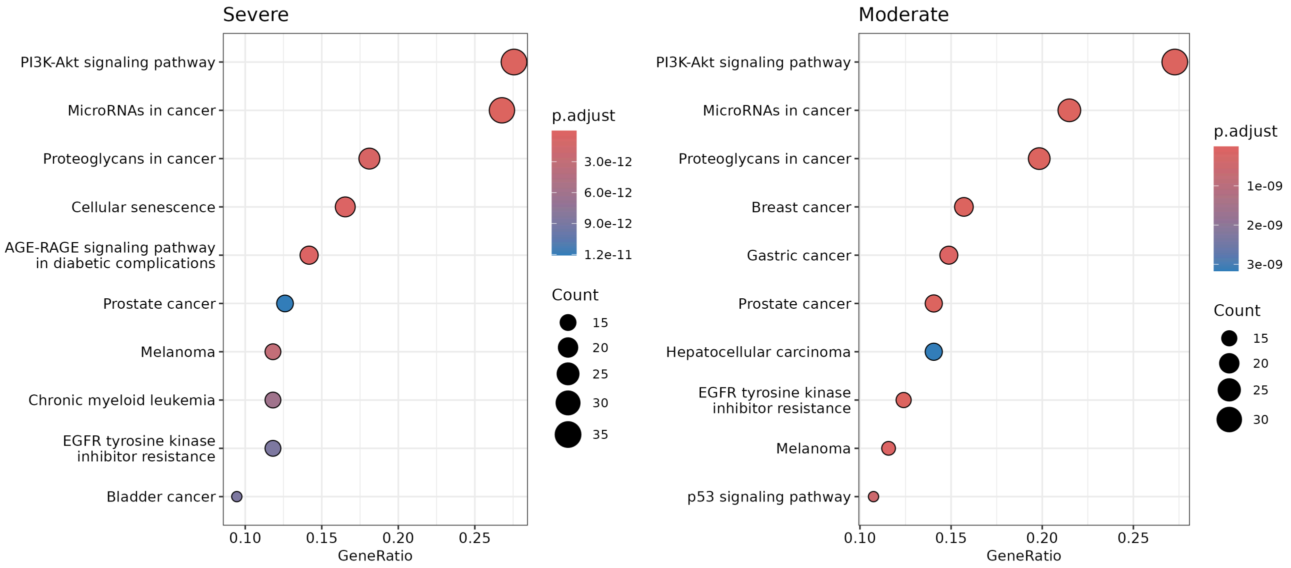

Supplementary Figure 4. Dotplot depicting KEGG pathway enrichment analysis results of the experimentally validated targets of consistenly differentially expressed miRNA families in patients with severe symptoms (T1 and T2) and patients with moderate symptoms (T1 and T2). Severe and moderate results are showed separatedly as the specific forms of hsa-let-7 and hsa-miR-16 differentially expressed in severe and moderate cases differ. Each point represents a KEGG pathway, with the color indicating statistical significance, the x-axis representing geneRatio, and point size correlating with gene count. Significantly enriched pathways are highlighted against a background of non-enriched pathways, based on an adjusted p-value threshold of 0.05

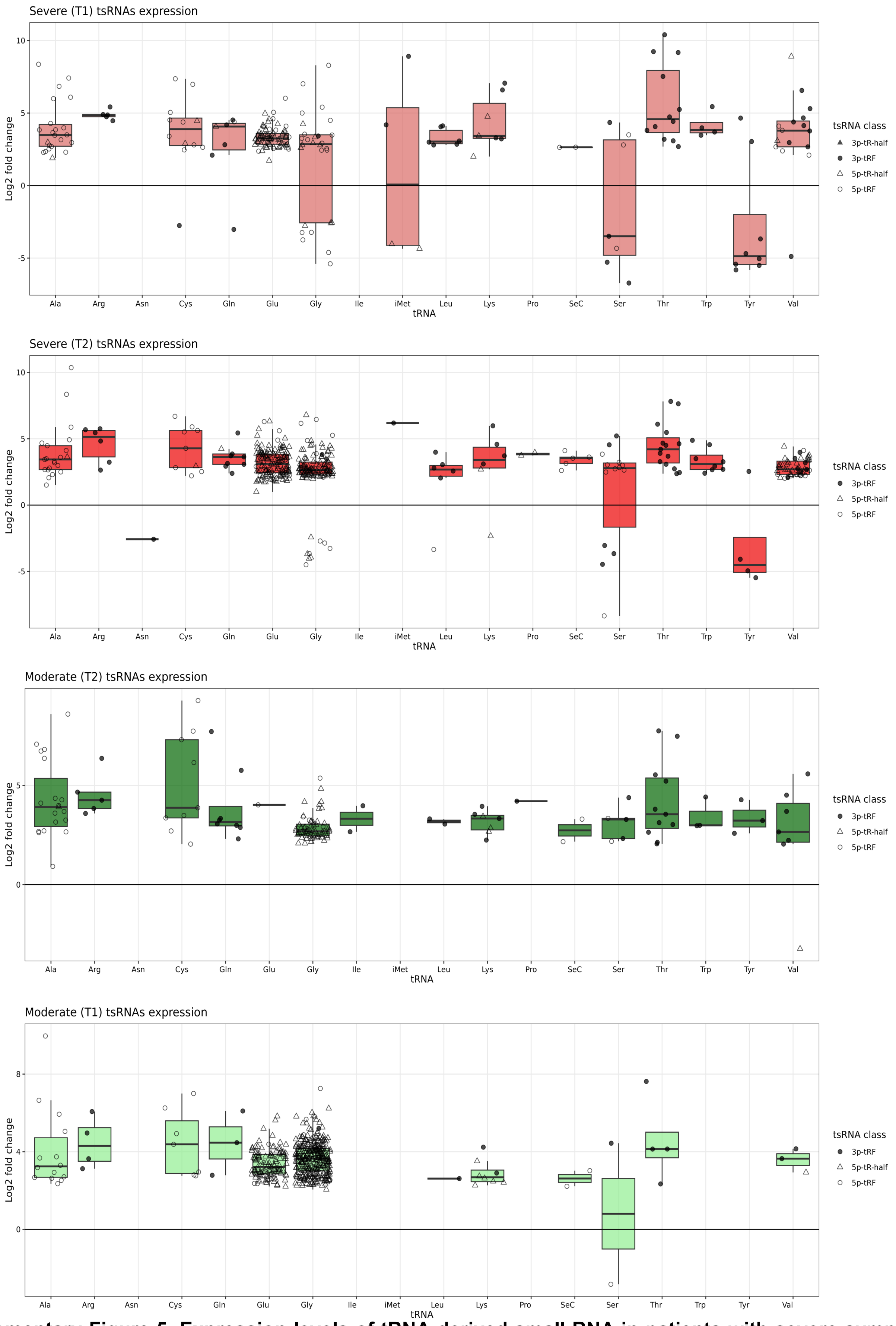

Supplementary Figure 5. Expression levels of tRNA-derived small RNA in patients with severe symptoms (T1 and T2) and patients with moderate symptoms (T1 and T2). Each point represents the expression value (log2FC) of a sequence in infected patients versus non-infected controls. Four classes of tRNA-derived small RNAs are represented: 5’ tRF, 3’ tRF, 5’ tR-half and 3’ tR-half. The different classes are depicted by distinctive symbols. In boxplots, the central lines depict the median, while the box boundaries represent the upper and lower quartiles. The whiskers extend to the first or last data point within 1.5x the interquartile range of the box boundaries in the lower and upper directions, respectively.

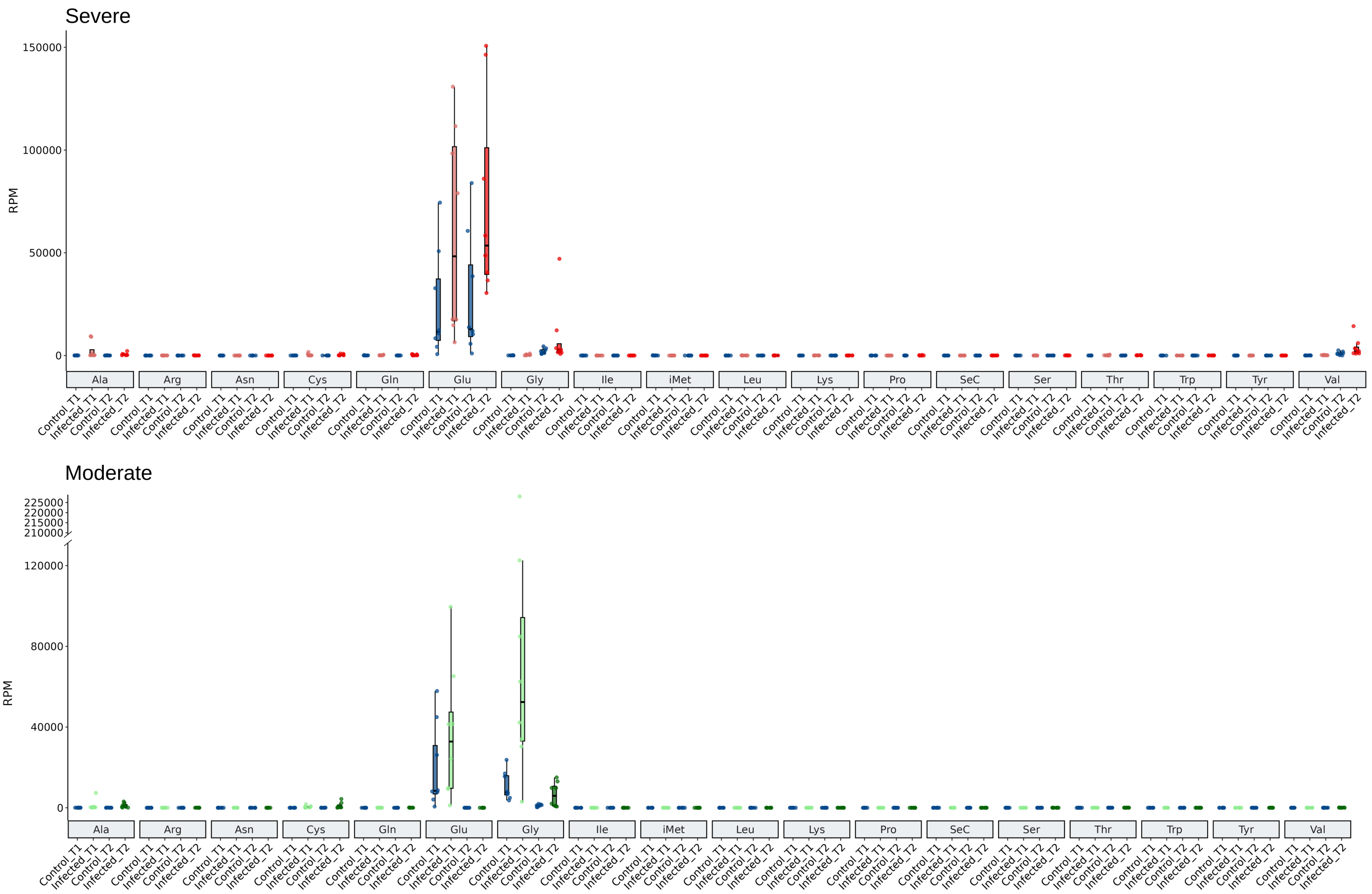

Supplementary Figure 6. Relative accumulation of the differentially expressed tRNA-derived sequences in patients with severe symptoms (T1 and T2) and patients with moderate symptoms (T1 and T2). Dots indicate the absolute accumulation in reads per million (RPM) of differentially expressed tsRNA sequences in control and infected simples for each condition (severe/moderate symptoms, T1/T2 time). Only the four main classes of tsRNAs are considered for the analysis: 5' tRF, 3' tRF 5' tR-half and 3' tR-half. In boxplots, the central lines depict the median, while the box boundaries represent the upper and lower quartiles. The whiskers extend to the first or last data point within 1.5x the interquartile range of the box boundaries in the lower and upper directions, respectively.

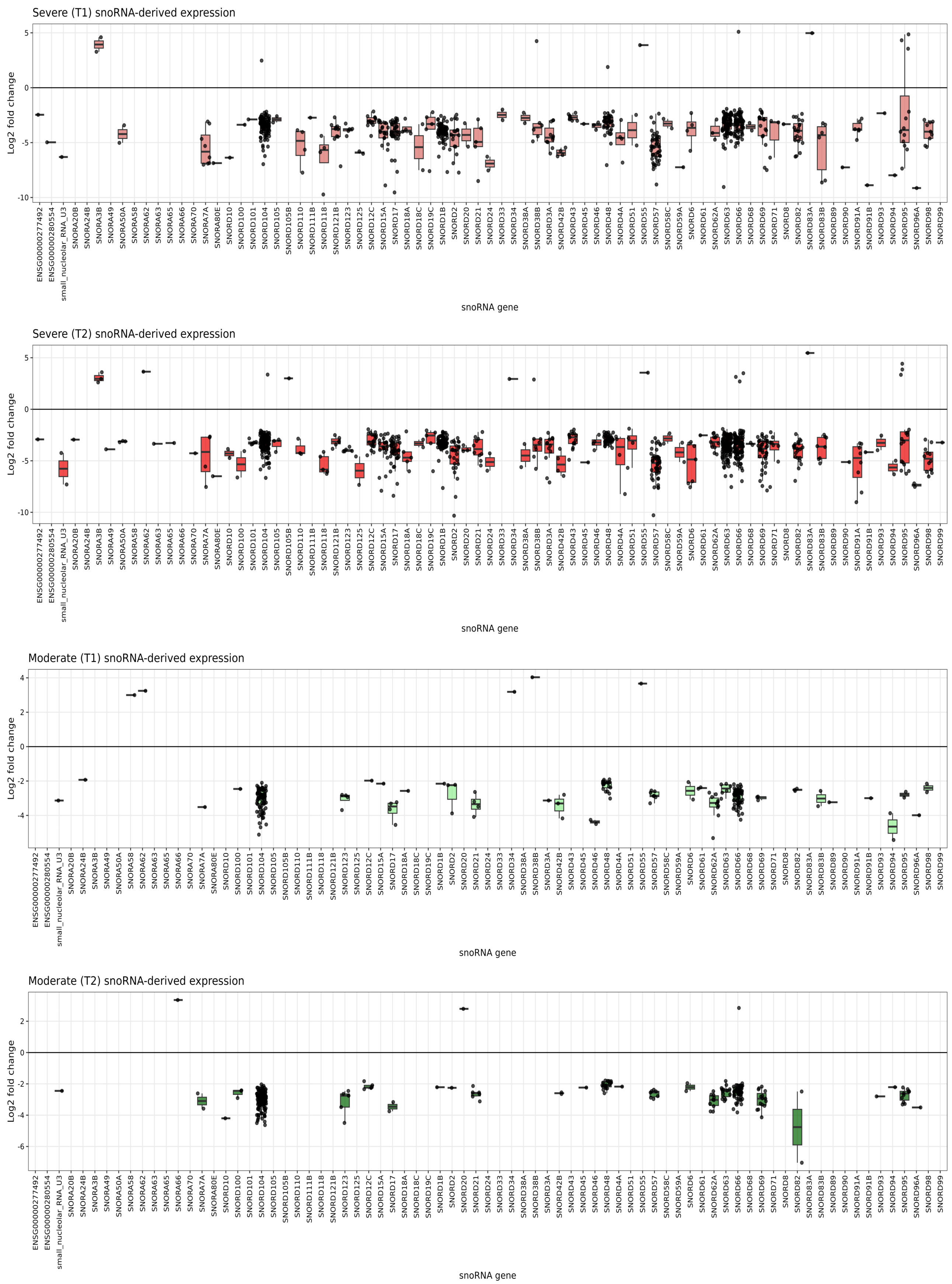

Supplementary Figure 7. Expression levels of snoRNA-derived small RNA in patients with acute symptoms (T1 and T2) and patients with mild symptoms (T1 and T2). Boxplot of the expression of each *snoRNA*. Each point represents the expression value (log2FC) of a sequence in infected patients versus non-infected controls. In boxplots, the central lines depict the median, while the box boundaries represent the upper and lower quartiles. The whiskers extend to the first or last data point within 1.5x the interquartile range of the box boundaries in the lower and upper directions, respectively.

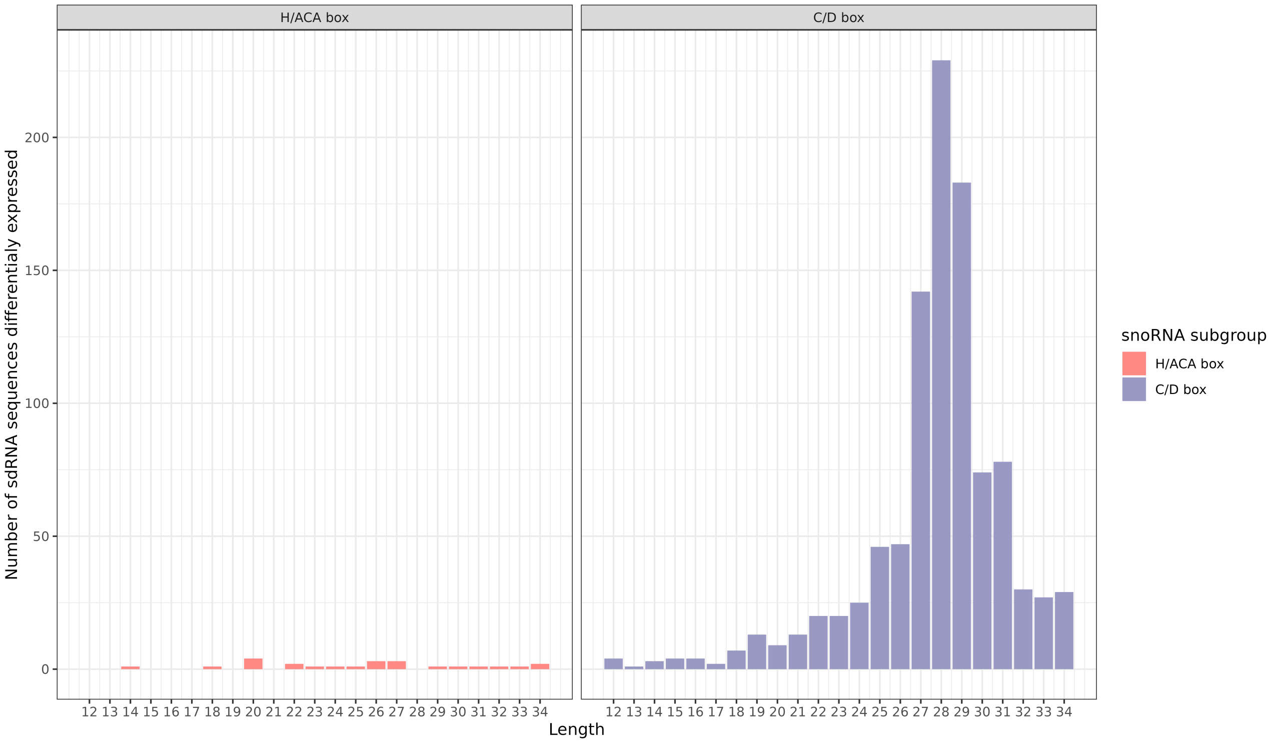

Supplementary Figure 8. Length distribution of differentially expressed sequences classified as snoRNA-derived. Histograms of the length distribution are represented separately depending on the subgroup of the parental snoRNA (H/ACA box or C/D box).

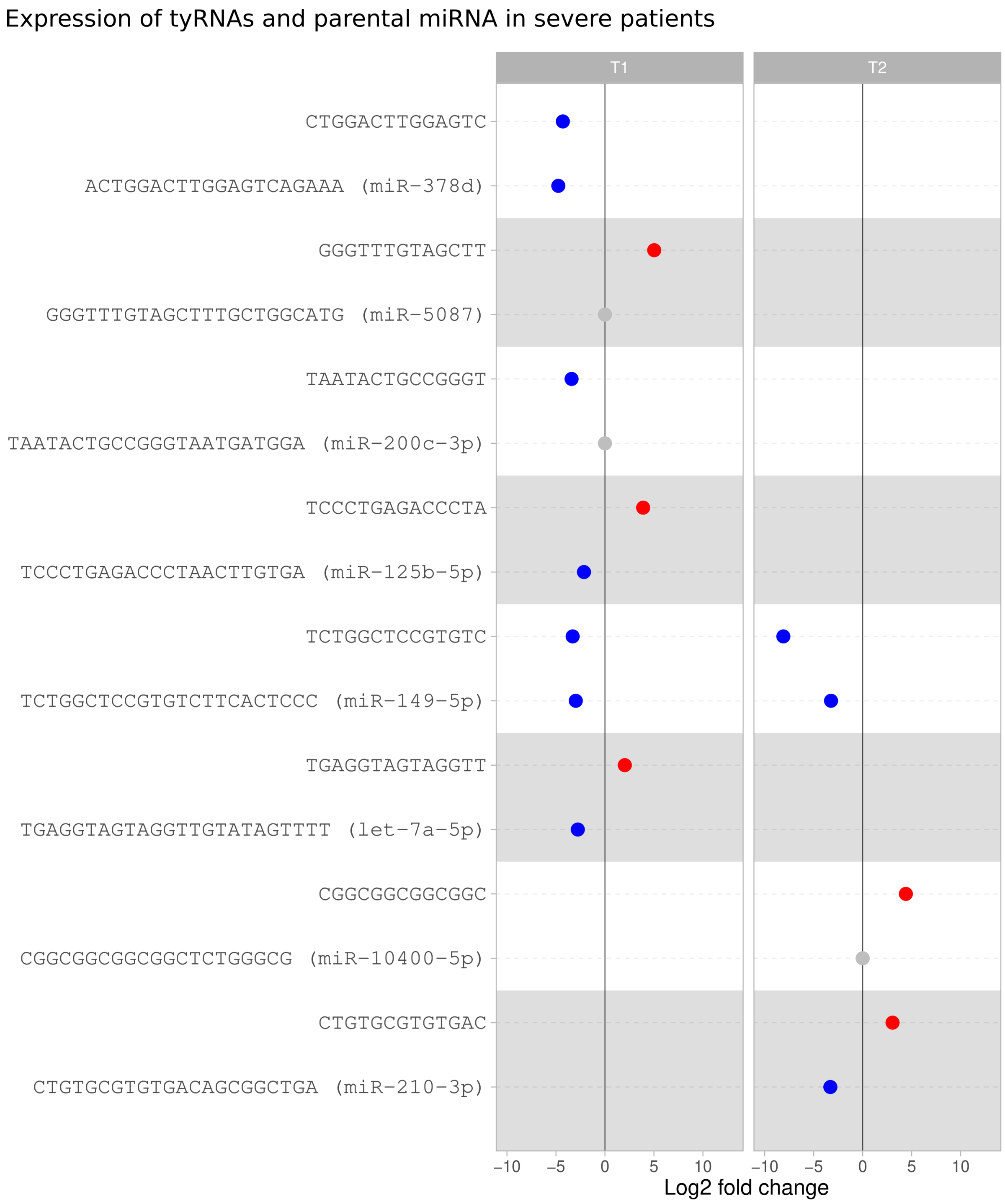

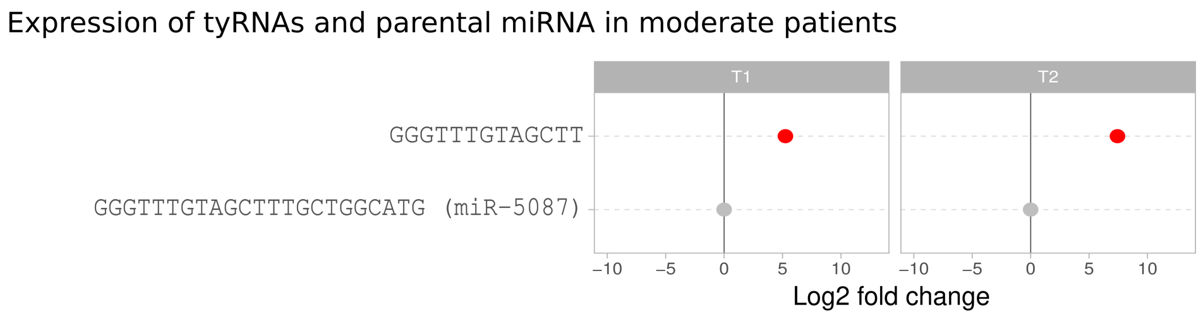

Supplementary Figure 9. Expression levels of tyRNAs and parental miRNAs in patients with severe symptoms (T1 and T2) and patients with moderate symptoms (T1 an T2). Each point represents the expression value (log2FC) of a sequence in infected patients versus *non-infected* controls. Expression values of the parental miRNAs are represented below the tyRNA value for each case.
