# Supplementary material for: SARS-CoV-2 remodels the landscape of small non-coding RNAs with infection time and symptom severity
Source: NPJ Syst Biol Appl. 2024 Apr 17;10:41. doi: 10.1038/s41540-024-00367-z (PMC11024147; doi:10.1038/s41540-024-00367-z)
Supplement: Supplementary file 12 — Supplementary Table 10 [file 41540_2024_367_MOESM12_ESM.pdf]

Supplementary Table 10: Validation of sncRNA accumulation stem loop by RT-qPCR

sRNA	Analyzed sRNA sequence	Diferential expression (Log2FC)			
		Sequencing	FDR	RT-qPCR	p value
Hsa-miR-16-2-3p	ACCAATATTACTGTGCTGCTTT	3,175	< 0.05	0,930	0,0316
Hsa-miR-16-1-5p	TAGCAGCACGTAAATATTGGC	2,405	< 0.05	1,582	8,18E-06
Glu-tsRNA	TCCCTGTGGTCTAGTGGTTAGGATTCGGCGCT	1,8	< 0.05	1,848	0,0002
Gly-tsRNA	GCATGGGTGGTTCAGTGGCAGAATTCTCGCCT	3,535	< 0.05	2,493	0,0003
SNORD55	GTGTATGATGACAACTCGGTAAT	3,71	< 0.05	0,394	0,0004

Additional information: The table shown the differential expression values of five representatives sncRNAs estimated by stem loop RT-qPCR from 3 independent SARS-CoV-2 infected patients and the values obtained for the same sequences by sRNA-seq analysis. The analyzed nasopharyngeal samples analyzed in this study (including uninfected controls) were collected in July 2021 at the Hospital Clínico Universitario de Valencia in Spain (as described in the Methods section), and were identified as SARS-CoV-2 infected by RT-qPCR (as described in the Methods section) with CT values of 17.68; 14.41 and 20.38. Considering that the sncRNAs selected for validation were not restricted to any specific time of infection or disease status, for this analysis we selected samples only classified as infected and uninfected.

For this analysis we selected sequences with significant differential expression (FDR < 0.05) in at least two of the four analyzed conditions (T1 and T2 for Severe and Moderate symptoms). As is showed, the selected sncRNAs identified as overexpressed by sequence analysis were also identified as up-regulated by stem loop RT-qPCR. The differential expression values represented correspond to the median of the $\log_2 FC$ values obtained using DESeq2 for each sncRNA. The Log2FC values for RT-qPCR correspond to the media of the values obtained from the three analyzed patients. The p-values for RT-qPCR were estimated by paired T-test.
